# Patients with diabetic retinopathy have high retinal venous pressure

**DOI:** 10.1186/s13167-015-0027-1

**Published:** 2015-02-24

**Authors:** Anna K Cybulska-Heinrich, Michael Baertschi, Cay Christian Loesche, Andreas Schoetzau, Katarzyna Konieczka, Tatjana Josifova, Josef Flammer

**Affiliations:** Department of Ophthalmology, University of Basel, Mittlere Strasse 91, CH-4031 Basel, Switzerland; Department of Ophthalmology, Hospital of Mülheim an der Ruhr, DE-45468 Mülheim an der Ruhr, Germany

**Keywords:** Diabetic retinopathy (DR), Contact lens dynamometer (CLD), Ocular dynamic force (ODF), Retinal venous pressure (RVP), Spontaneous venous pulsation, Ophthalmodynamometry, Personalized prevention

## Abstract

**Background:**

The introduction of ophthalmodynamometric measurement of retinal venous pressure (RVP) now permits the quantification, or at least an approximation, of the real pressure in the retinal veins.

**Methods:**

We measured the RVP of healthy control subjects, patients with diabetes without diabetic retinopathy (nonDR) and patients with diabetes and diabetic retinopathy (DR).

**Results:**

The mean ± SD RVP for the control, nonDR and DR groups were 23.4 ± 7.33, 22.5 ± 5.78 and 37.7 ± 10.1 mmHg, respectively. In the diabetes patients with DR, the RVP was markedly and significantly increased, and this result was significantly age dependent. RVP was not increased in the group of diabetes patients without DR. In our tested population, diabetes had a minor influence on intraocular pressure.

**Conclusion:**

Regardless of the cause, a marked increase in RVP in diabetes patients with DR is clinically relevant, as it reduces perfusion pressure and increases transmural pressure. The reduced perfusion pressure contributes to hypoxia, and the increased transmural pressure can facilitate retinal edema.

Diabetes is an increasing burden, and DR is one of its most severe complications. Strategies to recognize the risk for DR and to develop personalized prevention and therapy therefore have major implications.

**Trial registration:**

ClinicalTrials.gov ID: NCT01771835.

## Overview

Diabetic retinopathy (DR) is one of the leading causes of blindness and visual impairment [1]. Nonproliferative diabetic retinopathy (NPDR) often progresses to proliferative diabetic retinopathy (PDR), leading to severe visual impairment.

Hyperglycemia and disease duration are the main risk factors for both the incidence of NPDR and the conversion to PDR [2]. However, other factors, such as systemic arterial hypertension and dyslipidemia, also play a crucial role [3,4].

Although diabetes mellitus (DM) is primarily an endocrinal disease, it also affects both the large and small vessels in different parts of the body. Thus, diabetic complications are broadly classified as microvascular (retinopathy, nephropathy and diabetic foot) and macrovascular (heart disease, stroke and peripheral arterial disease) complications [5].

In clinical settings, visual acuity, fundus examination, optical coherence tomography (OCT) and fluorescein angiography (FA) are routinely used to diagnose DR and to monitor its progression. The retinal vascular system is of particular interest for its early involvement and easy accessibility. Several studies [6-8] have shown changes in retinal vessel calibre and geometry, depending on diabetes duration and severity. However, retinal venous pressure (RVP) has not been measured in patients with diabetes thus far. Until recently, RVP was assumed to be equal to intraocular pressure (IOP) [9], except under conditions of increased cerebrospinal fluid pressure [10]. The introduction of ophthalmodynamometric measurement of RVP presents new opportunities to expand ocular circulatory diagnostics.

Indeed, RVPs higher than IOPs have been observed under several conditions [9,11,12]. On average, RVP is elevated in patients with glaucoma [13,14] and in patients with retinal vein occlusion, to a greater extent in the ischemic than in the nonischemic type [15].

An increase in RVP is of major relevance, as it decreases ocular perfusion pressure (the difference between central arterial pressure and central RVP) [9,14] and increases transmural pressure, thus altering retinal fluid homeostasis, which contributes to retinal edema.

We tested the hypotheses that a) RVP in DM patients is different from normal controls and b) RVP in DM patients with and without retinopathy is different.

## Methods

This study was carried out at the eye clinic at the University of Basel. The protocol was approved by the institutional ethics committee of the Medical Faculty of the University of Basel. Each subject signed an informed consent form before entering the study.

### Subjects

We measured RVP a) in 254 eyes of 127 healthy control subjects, b) in 40 eyes of 20 patients with diabetes without diabetic retinopathy (nonDR) and c) in 54 eyes of 27 patients with diabetes and diabetic retinopathy (DRP). We did not distinguish between proliferative and nonproliferative DR, nor did we stage the severity of DR. Most of the patients with diabetes were under the care of internists at the Medical Faculty of the University of Basel, and the blood sugar, blood pressure and lipids of all the patients were well controlled.

The healthy controls were not on any medication. Exclusion criteria for all three groups were any additional eye disease; IOP values were not an exclusion criterion. By chance, the lowest IOP value was less than 10 mmHg and the highest value was more than 20 mmHg. The subjects with IOP above 20 mmHg showed no signs of glaucomatous optic neuropathy.

### Test procedure

We had conducted a pilot study in which the RVP of all the subjects was measured by two investigators, and the averages of the resulting values were identical. For this reason, the subjects included in the present study were measured by one of the two investigators. Both eyes of each subject were examined by a trained ophthalmologist to determine whether the subject qualified for inclusion in the study. On the day of the study, IOP was measured with a noncontact tonometer, after which the pupils were dilated with Tropiphen® (tropicamide 5 mg and phenylephrine 10 mg) eye drops. The central and hemiretinal veins on the disc surface were examined for the presence of spontaneous pulsation. If pulsation was observed, RVP was taken as equal to IOP. If no venous pulsation was observed, RVP was measured after the application of one drop of Alcaine® (proxymetacaine hydrochloride 5 mg, Alcon) for topical anesthesia.

### Measurement of RVP

A contact lens dynamometer (CLD) was used to measure RVP. The FDA-approved CLD (Meditron GmbH, Voelklingen, Germany) consists of a three-mirror Goldmann lens with a ring-shaped attachment containing several precision sensors affixed to the rear. The sensors continuously measure the force that the ophthalmologist exerts on the eye by means of the contact lens. The CLD is connected by a thin, flexible cable to a central unit approximately the size of a handheld calculator. A liquid crystal display shows the delta pressure in millimetres mercury, based on a calibration curve. The CLD, along with a contact fluid (Methocel® 2%; CIBA Vision, Germany), was placed on the cornea. Pressure was increased until one of the segments of veins on or close to the optic nerve head started to pulsate; the required pressure is called ocular dynamic force (ODF). The RVP was calculated as the sum of the ODF and the IOP [16]. If spontaneous venous pulsation was present, then the ODF was said to be 0. The measurements were conducted with a slit lamp biomicroscope (Haag-Streit) set at × 16 magnification.

The term RVP used herein refers to the pressure in the retinal veins close to the optic nerve and not generally in the retinal veins.

### Statistical analysis

Descriptive statistics of all the eyes are presented as means and SD or counts and percentages for all study groups. For age, the overall *p*-value was calculated using analysis of variance (ANOVA) for independent groups. For RVP and IOP, the overall *p*-values were calculated using ANOVA based on linear mixed effects models. For gender, *p*-value was based on the chi-square test. To assess the differences in RVP and IOP among the study groups, a linear mixed effects model was performed. Mixed effects models are suitable techniques to account for multiple correlated measurements within a subject.

The dependent variables were RVP or IOP, and the independent variables were study group, age and eye side. Thus, the results were adjusted for age and eye side. Subject ID is treated as a random factor to account for potential correlation between the left and right eyes. The results are presented as differences of the mean between the two diabetes groups and the controls with corresponding 95% confidence intervals and *p*-values.

In addition, the dependence of RVP on age was estimated for each study group using a nested mixed effects model and expressed as a regression slope.

The results are presented both without and with age matching. To balance the age distribution between the DR group and the controls, a propensity score matching [17] was performed. To avoid further loss of sample size, the nonDR group was not matched.

The dependence of RVP and IOP on age in the matched and unmatched study groups is presented graphically. A *p*-value <0.05 was considered significant. All analyses were conducted using the R statistical software package, version 2.15.1 [18]. Mixed effects models were performed using the “nlme” package.

## Results

**Figure 1:**
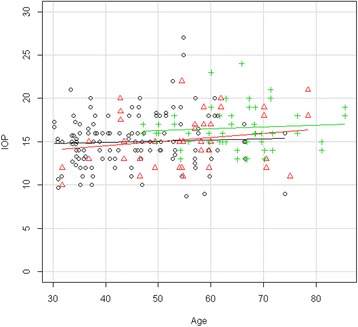
**Dependence of IOP on age for each study group (unmatched data).** Scatter plot of the IOP (mmHg) of each study group (unmatched data) plotted versus the age (years) of the subjects. Straight lines indicate regression slopes. Black circle control group, red triangle nonDR group, green cross DR group. In all of the groups, IOP had only a very slight tendency to increase with age.

**Figure 2:**
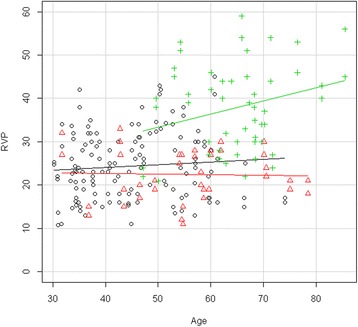
**Dependence of RVP on age for each study group (unmatched data).** Scatter plot of the RVP (mmHg) of each study group (unmatched data) plotted versus the age (years) of the subjects. Straight lines indicate regression slopes. Black circle control group, red triangle nonDR group, green cross DR group. RVP increased markedly in patients with DR, but not in diabetes patients without DR.

The demographic description of the entire population tested is provided in Table 1. The corresponding description of the age-matched population is provided in Table 2. Considering the total population tested, IOP tended to be slightly, but significantly, higher in the DR group than in the control group and the nonDR group (Table 1, Figure 1). RVP was clearly and significantly higher in the DR group than in the control group and the nonDR group (Table 3, Figure 2).

**Table 1:** **Descriptive statistics of the entire study population**

	**Control**	**NonDR**	**DR**	***p*** **overall**
	***N*** **= 254**	***N*** **= 40**	***N*** **= 54**	
Age	35.6 (13.4)	55.3 (12.3)	64.6 (9.09)	<0.001
IOP	15.1 (3.01)	15.3 (3.27)	16.6 (2.67)	<0.001
RVP	23.4 (7.33)	22.5 (5.78)	37.7 (10.1)	<0.001
Gender				0.002
Female	154 (60.6%)	12 (30.0%)	16 (29.6%)	
Male	100 (39.4%)	28 (70.0%)	38 (70.4%)	

The retinal venous pressure (RVP) and intraocular pressure (IOP) are given in millimetres mercury, and the age in years. They are expressed as the mean ± SD for each study group.

**Table 2:** **Descriptive statistics of age-matched data**

	**Control**	**NonDR**	**DR**	***p*** **overall**
	***N*** **= 28**	***N*** **= 40**	***N*** **= 28**	
Age	58.5 (7.28)	55.3 (12.3)	58.6 (7.02)	0.55
IOP	14.9 (3.18)	15.3 (3.27)	15.9 (2.27)	0.319
RVP	25.2 (9.11)	22.5 (5.78)	33.8 (9.81)	0.0076
Gender				0.56
Female	14 (50%)	12 (30.0%)	10 (35.7%)	
Male	14 (50%)	28 (70.0%)	18 (64.3%)	

The retinal venous pressure (RVP) and intraocular pressure (IOP) are given in millimetres mercury, and the age in years. They are expressed as the mean ± SD for each study group.

**Table 3:** **Mean differences in IOP and RVP between study groups for matched data**

	**Lower 95 CI**	**Difference of means**	**Upper 95 CI**	***p*** **value**
IOP				
NonDR–control	−1.72	0.18	2.09	0.85
DR–control	−0.72	1.33	3.38	0.20
DR–nonDR	−0.62	1.15	2.91	0.20
RVP				
NonDR–control	−6.33	−0.70	4.92	0.80
DR–control	0.30	6.37	12.44	0.040
DR–nonDR	2.37	7.07	11.77	0.004

The frequency of spontaneous venous pulsation was 24% in the control group, 25% in the nonDR group and 0% in the DR group.

**Figure 3:**
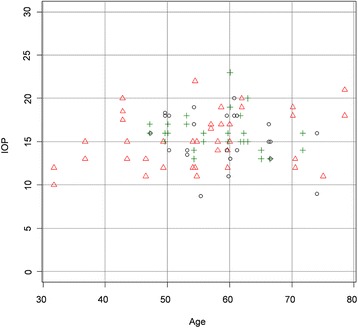
**Dependence of IOP on age for each study group (age-matched data).** Scatter plot of the IOP (mmHg) of each study group (matched data) plotted versus the age (years) of the subjects. Regression line is not shown as the slope was not significantly different from zero. Black circle control group, red triangle nonDR group, green cross DR group.

**Figure 4:**
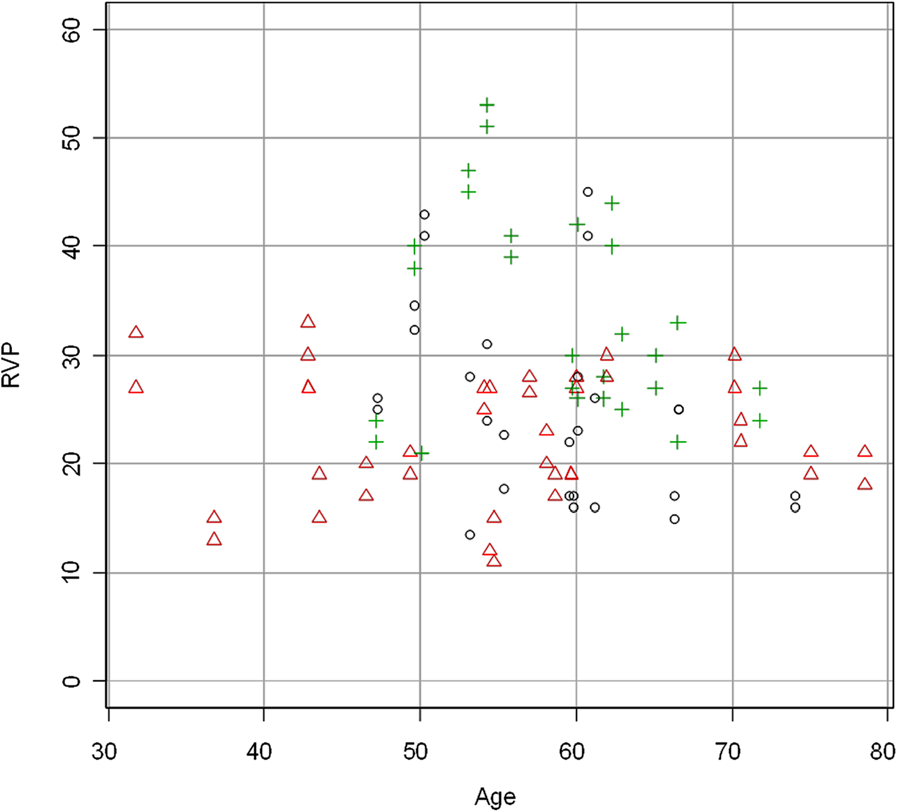
**Dependence of RVP on age for each study group (age-matched data).** Scatter plot of the RVP (mmHg) of each study group (matched data) plotted versus the age (years) of the subjects. Regression line is not shown as the slope was not significantly different from zero. Black circle control group, red triangle nonDR group, green cross DR group.

The patients with diabetes, particularly the ones with DR, were significantly older (Table 1). Therefore, a second analysis was performed on age-matched data. While IOP was now not significantly different between the groups anymore (Figure 3), RVP in these smaller groups still remained significantly higher in the patients with DR than in the control group and the nonDR group (Table 2, Figure 4). In other words, RVP was higher in the DR group compared to both the control and nonDR groups (Table 3).

There was a highly significant dependence of RVP on age for the nonmatched DR group (*p* < 0.001, slope = 0.52, 0.26–0.78). All other matched and nonmatched dependencies on age were not significant.

## Discussion

We tested the hypothesis that a) RVP in DM patients is different from normal controls and b) RVP in DM patients with and without retinopathy is different.

In our tested population, the RVP of the group of diabetes patients without DR was not higher than that of the control group (Table 2). The reason RVP was slightly lower in the nonDR group remains unknown.

The RVP of the diabetes patients with DR was markedly and significantly higher than that of the control subjects and the diabetes patients without DR (Table 3). This increase was significantly age dependent (Figure 2).

In addition, we found in our tested population that diabetes had a minor influence on IOP (Table 1).

Limitations of the study include lack of blood pressure measurement, lack of differentiation among types of diabetes, unknown duration of diabetes and gender differences between the control and DR groups. Theoretically, the higher RVP in the group of patients with DR might be caused by different blood pressures in the groups. However, this is very unlikely for the following reasons. 1) There is no indication in the literature that RVP is blood pressure dependent [14,19]. 2) According to the treating internist, the blood pressure of all of the diabetes patients was well controlled. The blood pressure was measured in a timely fashion (within 10 days), and the values never exceeded 150/90 mmHg. If we missed arterial hypertension, it was in the control group, where we relied on information from the participants.

A number of questions remain open. We do not know whether the increase in RVP with age reflects the duration of the disease or stage of severity. Furthermore, we do not know the cause of the RVP increase. It could be due to structural changes in the veins or the structures around the veins [20], or it could reflect a dysregulation of the retinal veins in the area of the optic nerve head or lamina cribrosa [21].

Vascular and hemodynamic changes in the retina of patients with DM are well known and have often been reported [21]. While blood flow velocity in the retinal arterioles and venules of DM patients with nonproliferative diabetic retinopathy is decreased [22], it is increased patients with early DM without DR [23].

Diabetes also leads to variations in retinal vascular calibre and in particular to a widening of retinal venular calibre in patients with DR [24,25]. In these studies, RVP was neither measured nor do their outcomes explain the increase in RVP.

Future studies are required to confirm the increase in RPV in patients with DR. The study design should consider other parameters as well, such as blood pressure, age, type and stage of diabetes and therapy.

Regardless of the cause, a marked increase in RVP in diabetes patients with DR is clinically relevant, as it reduces perfusion pressure and increases transmural pressure. The reduced perfusion pressure contributes to hypoxia, and the increased transmural pressure facilitates retinal edema; hypoxia and edema are major components of DR. While increased RVP contributes to retinal hypoxia, such local hypoxia might increase RVP, thus inducing a vicious circle [26].

### Outlook

Clinical and scientific implications of our findings are at present still open. Further studies are needed to establish the relationship between RVP and different factors such as blood pressure, duration of diabetes, or HB1c level. We also must compare diabetes type 1 with diabetes type 2 and NPDR with PDR. Of special interest is the question whether an increased RVP predicts the development of a future DR and whether it modifies the course of an existing DR. We like to know the relationship between RVP and retinal hemorrhages and whether an increased RVP predisposes to a retinal vein occlusion. Of special interest is the question whether we can reduce RVP and if so, whether this may help to prevent or modify DR. If this will be the case, we will be one step further in the direction of a predictive, preventive and personalized medicine.
